# Genetic diversity is a predictor of mortality in humans

**DOI:** 10.1186/s12863-014-0159-7

**Published:** 2014-12-29

**Authors:** Nathan A Bihlmeyer, Jennifer A Brody, Albert Vernon Smith, Kathryn L Lunetta, Mike Nalls, Jennifer A Smith, Toshiko Tanaka, Gail Davies, Lei Yu, Saira Saeed Mirza, Alexander Teumer, Josef Coresh, James S Pankow, Nora Franceschini, Anish Scaria, Junko Oshima, Bruce M Psaty, Vilmundur Gudnason, Gudny Eiriksdottir, Tamara B Harris, Hanyue Li, David Karasik, Douglas P Kiel, Melissa Garcia, Yongmei Liu, Jessica D Faul, Sharon LR Kardia, Wei Zhao, Luigi Ferrucci, Michael Allerhand, David C Liewald, Paul Redmond, John M Starr, Philip L De Jager, Denis A Evans, Nese Direk, Mohammed Arfan Ikram, André Uitterlinden, Georg Homuth, Roberto Lorbeer, Hans J Grabe, Lenore Launer, Joanne M Murabito, Andrew B Singleton, David R Weir, Stefania Bandinelli, Ian J Deary, David A Bennett, Henning Tiemeier, Thomas Kocher, Thomas Lumley, Dan E Arking

**Affiliations:** Predoctoral Training Program in Human Genetics, McKusick-Nathans Institute of Genetic Medicine, Johns Hopkins University School of Medicine, Baltimore, MD USA; McKusick-Nathans Institute of Genetic Medicine, Johns Hopkins University School of Medicine, BRB Room 447, 733 N. Broadway St, Baltimore, MD 21205 USA; Department of Statistics, University of Auckland, 303.325 Science Centre, Private Bag 92019, Auckland, 1142 New Zealand; Department of Pathology, University of Washington, Seattle, WA USA; Departments of Medicine, Epidemiology, and Health Services, University of Washington, Seattle, WA USA; Laboratory of Neurogenetics, National Institute on Aging, National Institutes of Health, Bethesda, MD USA; Laboratory of Epidemiology, Demography and Biometry, National Institute on Aging, National Institutes of Health, Bethesda, MD USA; Department of Epidemiology and Prevention, Division of Public Health Sciences, Wake Forest University School of Medicine, Winston-Salem, NC USA; Department of Biostatistics, Boston University School of Public Health, Boston, MA USA; The National Heart Lung and Blood Institute’s Framingham Heart Study, Framingham, MA USA; Section of General Internal Medicine, Department of Medicine, Boston University School of Medicine, Boston, MA USA; Institute for Aging Research, Hebrew Senior Life, Department of Medicine, Beth Israel Deaconess Medical Center and Harvard Medical School, Cambridge, MA USA; Survey Research Center, Institute for Social Research, University of Michigan, Ann Arbor, MI USA; Department of Epidemiology, School of Public Health, University of Michigan, Ann Arbor, MI USA; Centre for Cognitive Ageing and Cognitive Epidemiology, The University of Edinburgh, Edinburgh, UK; Department of Psychology, The University of Edinburgh, Edinburgh, UK; Alzheimer Scotland Dementia Research Centre, The University of Edinburgh, Edinburgh, UK; Rush Alzheimer’s Disease Center, Rush University Medical Center, Chicago, IL USA; Program in Translational NeuroPsychiatric Genomics, Department of Neurology, Brigham and Women’s Hospital and Harvard Medical School, Boston, MA USA; Rush Institute for Healthy Aging and Department of Internal Medicine, Rush University Medical Center, Chicago, IL USA; Department of Epidemiology, Erasmus Medical Centre, Rotterdam, The Netherlands; Department of Neurology, Erasmus Medical Centre, Rotterdam, The Netherlands; Department of Radiology, Erasmus Medical Centre, Rotterdam, The Netherlands; Department of Child and Adolescent Psychiatry, Erasmus Medical Centre, Rotterdam, The Netherlands; Department of Psychiatry, Erasmus Medical Centre, Rotterdam, The Netherlands; Department of Internal Medicine, Erasmus Medical Centre, Rotterdam, The Netherlands; Interfaculty Institute for Genetics and Functional Genomics, University Medicine Greifswald, Greifswald, Germany; Institute for Community Medicine, University Medicine Greifswald, Greifswald, Germany; Department of Psychiatry and Psychotherapy, University Medicine Greifswald, HELIOS Hospital Stralsund, Greifswald, Germany; German Center for Neurodegenerative Diseases (DZNE), Site Rostock/Greifswald, Greifswald, Germany; Unit of Periodontology, Department of Restorative Dentistry, Periodontology and Endodontology, University Medicine Greifswald, Greifswald, Germany; Icelandic Heart Association, Kopavogur, Iceland; University of Iceland, Reykjavik, Iceland; National Institute on Aging, National Institutes of Health, Bethesda, MD USA; Cardiovascular Health Research Unit, Department of Medicine, University of Washington, Seattle, WA USA; Translational Gerontology Branch, National Institute on Aging, Baltimore, MD USA; Geriatric Unit, Azienda Sanitaria Firenze (ASF), Florence, Italy; Department of Epidemiology, Johns Hopkins Bloomberg School of Public Health, Baltimore, MD USA; Division of Epidemiology and Community Health, University of Minnesota, Minneapolis, MN USA; Department of Epidemiology, School of Public Health, University of North Carolina at Chapel Hill, Chapel Hill, NC 27514 USA

**Keywords:** Heterozygosity, Human, Survival, GWAS

## Abstract

**Background:**

It has been well-established, both by population genetics theory and direct observation in many organisms, that increased genetic diversity provides a survival advantage. However, given the limitations of both sample size and genome-wide metrics, this hypothesis has not been comprehensively tested in human populations. Moreover, the presence of numerous segregating small effect alleles that influence traits that directly impact health directly raises the question as to whether global measures of genomic variation are themselves associated with human health and disease.

**Results:**

We performed a meta-analysis of 17 cohorts followed prospectively, with a combined sample size of 46,716 individuals, including a total of 15,234 deaths. We find a significant association between increased heterozygosity and survival (P = 0.03). We estimate that within a single population, every standard deviation of heterozygosity an individual has over the mean decreases that person’s risk of death by 1.57%.

**Conclusions:**

This effect was consistent between European and African ancestry cohorts, men and women, and major causes of death (cancer and cardiovascular disease), demonstrating the broad positive impact of genomic diversity on human survival.

**Electronic supplementary material:**

The online version of this article (doi:10.1186/s12863-014-0159-7) contains supplementary material, which is available to authorized users.

## Background

With the advent of genome-wide association studies (GWAS), and more recently whole-exome and whole-genome sequencing, remarkable progress has been made in elucidating the genetics of complex traits, with numerous genetic variants each explaining a small fraction of the variance [1,2]. The presence of numerous segregating small effect alleles within the genome that influence traits that directly impact health raises the question of whether global measures of genomic variation are themselves associated with human health and disease. Indeed, increased fitness has been associated with the increase of genetic diversity across many organisms [3,4], including humans [5-8], and is often referred to as positive Heterozygosity Fitness Correlations (HFCs). In particular, associations have been found between heterozygosity at the Major Histocompatibility Complex (MHC) (a.k.a. Human Leukocyte Antigen, HLA) region and general health in humans [9]. In the case of heterozygosity in the MHC region, the cause of a positive HFC being observed is believed to be the result of increased antibody diversity conveying robust pathogen resistance and therefore increased general health [10]. However, in the case of increased whole-genome heterozygosity, the mechanism of action is less readily apparent. Two general mechanisms that act at a genome level to influence fitness have been proposed. The first is compensation for recessive deleterious mutations [11], whereas the second is a specific advantage of the heterozygous state over either homozygous state (overdominance/heterozygous advantage) [11], such as that observed for the sickle cell mutation in the presence of endemic malarial disease. It has been proposed that compensation for deleterious mutations occurs at many loci and is the major mechanism at work in HFCs, with overdominance occurring at few loci but with greater effect size per occurrence [11].

## Results and discussion

Various heterozygosity metrics have been proposed [12]. The heterozygosity metric used in this study is the sum of all heterozygous loci divided by the expected state given the allele frequency under Hardy-Weinberg Equilibrium $t=\frac{{\displaystyle \sum }0;1}{{\displaystyle \sum }2p\left(1-p\right)}$: where p is the frequency of the major allele in each cohort. This metric up-weights loci where the expectation of being heterozygous is low. Given the relationship between effect size and allele frequency [13,14], up-weighting loci with low minor allele frequencies should maximize the ability to detect a HFC in humans under a model in which the compensation for deleterious alleles is the major mechanism driving HFCs. Only Single Nucleotide Polymorphisms (SNPs) on the autosomes were considered.

**Figure 1:**
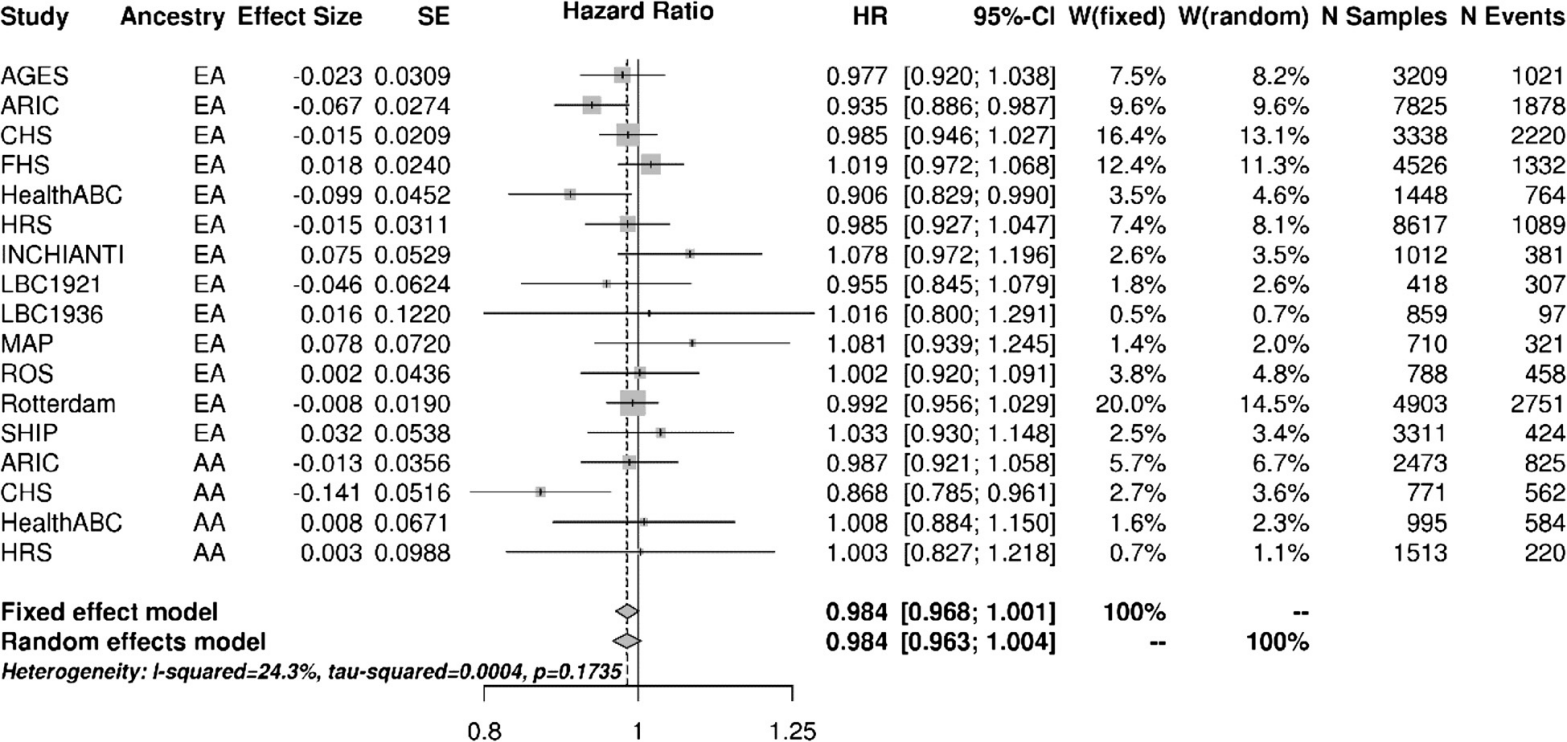
**Heterozygosity meta-analysis by study.** 1.57% decreased risk of death for every standard deviation increase in heterozygosity. This is determined using an inverse variance weighted fixed effect model. Significance of P = 0.03 is determined using Stouffer's method to combine Z-scores due to bias in inverse variance weighted fixed effect model. There are 46,716 individuals, including a total of 15,234 deaths. EA = European Ancestry; AA = African Ancestry; AGES = Age, Gene/Environment Susceptibility cohort; ARIC = Atherosclerosis Risk In Communities cohort; CHS = Cardiovascular Health Study; FHS = Framingham Heart Study; HealthABC = HealthABC cohort; HRS = Health and Retirement Study; INCHINTI = InCHIANTI cohort; LBC1921 = 1921 Lothian Birth Cohort; LBC1936 = 1936 Lothian Birth Cohort; MAP = Rush Memory and Aging Project cohort; ROS = Religious Orders Study; Rotterdam = Rotterdam Study; SHIP = Study of Health In Pomerania cohort; SE = Standard Error; HR = Hazard Ratio; CI = Confidence Interval; W = Weight; N = Number.

**Figure 2:**
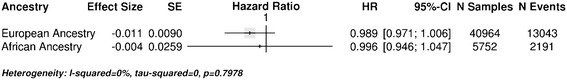
**Ancestry meta-analysis.** Direct comparison of European Ancestry to African ancestry cohorts showed no significant difference (P = 0.80). Figure is formatted the same as Figure 1.

To test for the effect of genome-wide heterozygosity on survival, we performed a meta-analysis of 17 cohorts (13 European ancestry, 4 African American ancestry) followed prospectively, with a combined sample size of 46,716 individuals, including a total of 15,234 deaths (Additional file 1: Table S1). Within each cohort, a Cox proportional hazards model (CoxPH) was used comparing age at study entry to age at study exit (death) or most recent follow-up (alive), and included covariates known to affect survival (sex, highest education level, Body Mass Index (BMI), income level, center where DNA was collected, and the first ten principal components to adjust for population substructure). Since each cohort used a different number of SNPs (Additional file 1: Table S1), the variances of the heterozygosity metrics are not the same (they are dependent on the total number of SNPs in the metric), and effect sizes from each cohort are not directly comparable. Using Stouffer's method to combine Z-scores, weighted by the number of deaths in each cohort, we find a significant association between increased heterozygosity and survival (P = 0.03). To assess effect size, we standardized the beta estimates by multiplying them by the standard deviation of the heterozygosity metric for each cohort [15]. This method does not completely account for the aforementioned bias; however, it is the most appropriate method to determine an interpretable effect size. Combining the standardized beta estimates using inverse variance weighting demonstrates that for every standard deviation increase in heterozygosity a person has over the population mean, they are expected to have a 1.57% decreased risk of death (Figure 1). There was no evidence for heterogeneity across studies, and a direct comparison of European Ancestry to African ancestry cohorts showed no significant difference (Figure 2, P = 0.80); thus, all downstream analyses combined European and African ancestry cohorts.

**Figure 3:**
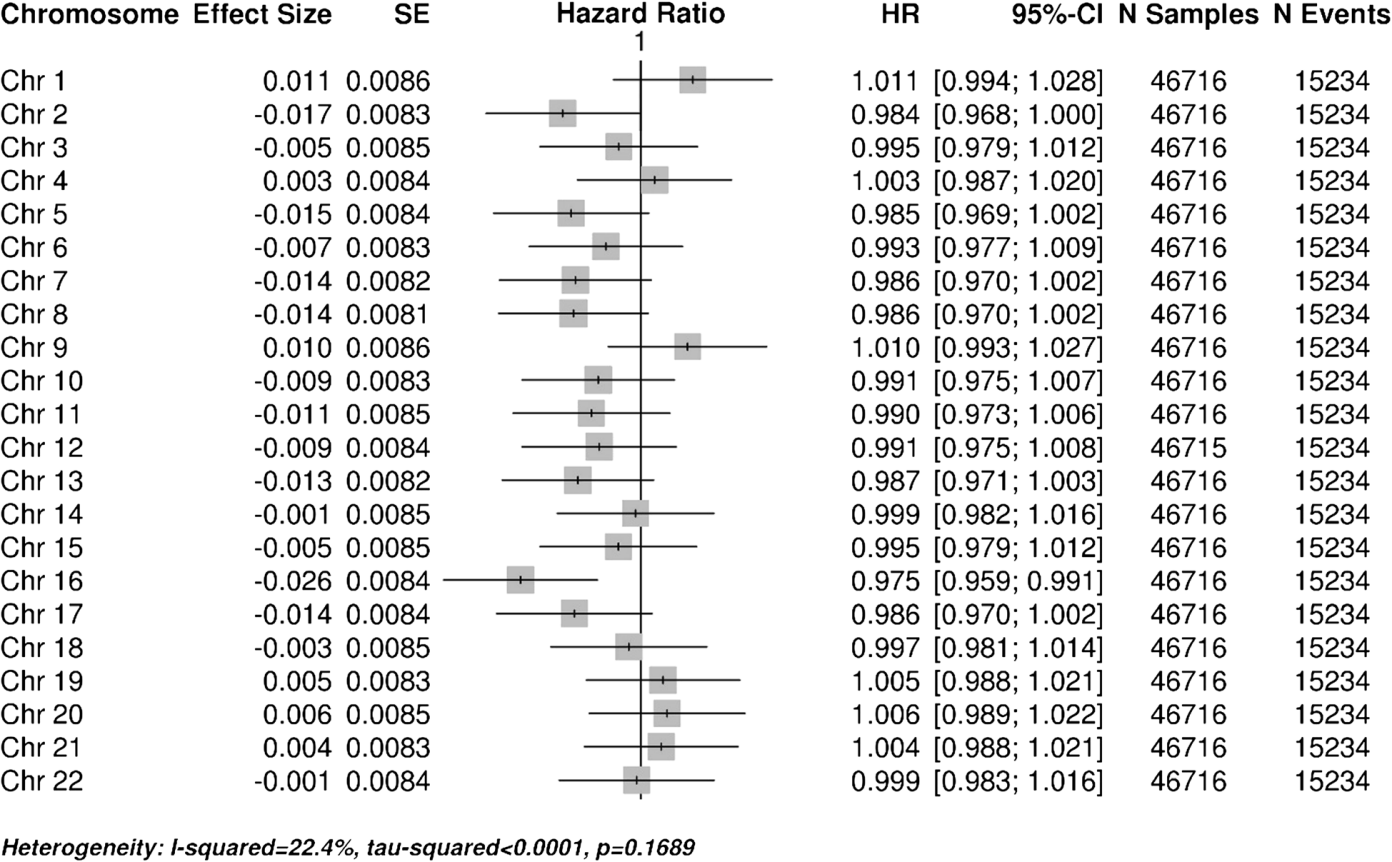
**Chromosome meta-analysis.** A meta-analysis for each chromosome was performed across studies. No significant difference was observed between effects across chromosomes (P = 0.17). Figure is formatted the same as Figure 1.

**Figure 4:**
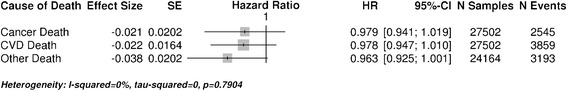
**Causes of death meta-analysis.** A meta-analysis for each cause of death was performed. Our results show no significant evidence for heterogeneity (Figure 4, P = 0.79). Figure is formatted the same as Figure 1.

**Figure 5:**
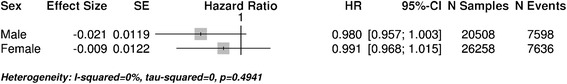
**Sex meta-analysis.** A meta-analysis was performed separately for each sex. Our results do not provide evidence for a differential effect of heterozygosity on survival in men vs. women (Figure 5, P = 0.49). Figure is formatted the same as Figure 1.

To test whether all chromosomes are contributing equally to the association between heterozygosity and survival, each study subject’s heterozygosity score was recalculated using only SNPs from a given chromosome. An inverse-variance meta-analysis for each chromosome was performed across studies, followed by a meta-analysis of the chromosomal results (Figure 3). No significant difference was observed between effects across chromosomes (P = 0.17). To test whether all major causes of death contribute equally to our genome-wide finding, death caused by cancer, death caused by CVD, and other causes of death were each analyzed separately. A meta-analysis for each cause of death was performed as described above, followed by a test for heterogeneity and model fitting. Our results demonstrate that heterozygosity is protective for all causes of death, with no significant evidence for heterogeneity (Figure 4, P = 0.79). To assess if heterozygosity levels impact women differently from men, meta-analyses were performed separately for each sex. Our results do not provide evidence for a differential effect of heterozygosity on survival in men vs. women (Figure 5, P = 0.49).

## Conclusions

In summary, this study provides evidence that the protective effect of increased heterozygosity seen in lower organisms functions in humans as well and may have implications for how we design future studies to identify genetic determinants of human disease and survival. We estimate that within a single population, every standard deviation of heterozygosity an individual has over the mean decreases that person’s risk of death by 1.57%. Interestingly, this seems to be true even if the population itself has reduced mean heterozygosity. In future studies, limiting to heterozygosity in proximity to genes and/or regulatory elements may reveal if some regions are more sensitive to heterozygosity than others. Increasing the African ancestry sample size may increase power to see a difference between ancestry groups. Overall the consistency we observed between European and African ancestry, males and females, and major causes of death demonstrate a broad positive impact of genomic diversity on human survival.

## Methods

Methods for each individual cohort can be found in Additional file 2: Text S1. Self-described Caucasian (“white”, “Caucasian”) and African ancestry (“black”, “African American”) individuals were included after excluding first and second degree relatives and genetic outliers. Genetic outliers were defined by merging genotyping data with HapMap3 data, and calculating the Euclidean distance from a combined reference HapMap3 population (Caucasian = CEU + TSI, African ancestry = ASW + YRI + MKK + LWK) cluster centroid in the first 3 PC space weighted by explained variance. Specifically, the standard deviation of Euclidean distance was determined for each HapMap reference group, and any sample greater than ten standard deviations away from centroid were defined as genetic outliers and excluded.

Directly genotyped SNPs were used for all analyses (Additional file 3: Figure S1). Imputed SNPs were not used to avoid issues with genotype accuracy and bias towards the reference panel. SNP exclusion criteria included: monomorphic in the dataset, non-unique mapping to Hg19, SNPs which are no longer in the company provided annotation file for the SNP array, >0.5% missing data, MAF ≤ 10%, HWE p-value ≥ 0.001, and non-autosomal SNPs. The heterozygosity metric is the sum of all heterozygous loci divided by the expected state given the allele frequency under Hardy-Weinberg Equilibrium: $t=\frac{{\displaystyle \sum }0;1}{{\displaystyle \sum }2p\left(1-p\right)}$ where p is the frequency of the major allele.

Separate association analyses were run for Caucasian and African ancestry samples from each cohort. The Cox Proportional Hazard Model (CoxPH) included covariates for Body Mass Index (BMI) at first visit and first ten principal components, and the 'strata' function for sex, education level (defined as 1. ≤11th grade, 2. high school diploma, general equivalence diploma or some vocational school, 3. 1–4 years of college, 4. Some graduate/professional school, and Missing), income level (defined by cohorts), and center of DNA collection within cohorts. The CoxPH model was set up so that the outcome was age at study entry, age at study exit, and a binary variable coding state of death (1: Dead, 0: Alive). Age is measured in units of years, but is accurate to the nearest day.

For the meta-analysis, significance was determined by Stouffer's method [16] calculated as a two-sided test by incorporating Z-scores derived from two-sided tests performed in each cohort. We standardized the beta estimates by multiplying them by the standard deviation of the heterozygosity metric for each cohort, to account for the fact that the effect size is proportional to the variance in the heterozygosity metric. The variance heterozygosity metric in turn is proportional to the inverse of the square root of the number of SNPs used to determine the heterozygosity metric. Because most cohorts used different genotyping arrays, a large bias is introduced into the meta-analysis. Stouffer’s method completely removes this bias; however, cannot estimate a combined effect size, only the overall significance. To get an estimate of the combined effect size (recognizing that the P-value and associated confidence intervals will be inflated), we used inverse variance weighting of the standardized cohort effect sizes, which partially corrects the bias and allows for the combined effect size to be estimated.

### Ethics statements

Institutional Review Board approvals were obtained by each participating ARIC study center (the Universities of NC, MS, MN, and John Hopkins University) and the coordinating center (University of NC), and the research was conducted in accordance with the principles described in the Helsinki Declaration. All subjects in the ARIC study gave informed consent. For more information see dbGaP Study Accession: phs000280.v2.p1. JHSPH IRB number H.34.99.07.02.A1. Manuscript proposal number MS1964.

HealthABC Human subjects protocol UCSF IRB is H5254-12688-11.

CHS was approved by institutional review committees at each site, the subjects gave informed consent, and those included in the present analysis consented to the use of their genetic information for the study of cardiovascular disease. It is the position of the UW IRB that these studies of de-identified data, with no patient contact, do not constitute human subjects research. Therefore we have neither an approval number, nor an exemption.

IRB permission to conduct genetics-related work in the Health and Retirement Study (HRS) is granted under the project title, "Expanding a National Resource for Genetic Research in Behavioral & Health Science" (HUM00063444). The IRB that approved this project is the Health Sciences and Behavioral Sciences Institutional Review Board at the University of Michigan. No manuscript proposal is required for use of HRS data.

Inchianti ethics review statement: The study protocol was approved by the Italian National Institute of Research and Care of Aging Institutional Review and Medstar Research Institute (Baltimore, MD).

The Religious Orders Study (ORA# 91020181) and the Rush Memory and Aging Project (ORA# 86121802) were approved by the Institutional Review Board of Rush University Medical Center. Written informed consent was obtained from all the participants.

The SHIP study followed the recommendations of the Declaration of Helsinki. The study protocol of SHIP was approved by the medical ethics committee of the University of Greifswald. Written informed consent was obtained from each of the study participants. The SHIP study is described in PMID: 20167617.

The Rotterdam Study has been approved by the medical ethics committee according to the Population Study Act Rotterdam Study, executed by the Ministry of Health, Welfare and Sports of the Netherlands. A written informed consent was obtained from all participants.

The Boston University Medical Campus Institutional Review Board approved the FHS genome-wide genotyping (protocol number H-226671) and genetic investigation of aging and longevity phenotypes (protocol number H-24912).

The Age, Gene/Environment Susceptibility Reykjavik Study has been funded by NIH contract N01-AG-12100, the NIA Intramural Research Program, Hjartavernd (the Icelandic Heart Association), and the Althingi (the Icelandic Parliament). The study is approved by the Icelandic National Bioethics Committee, (VSN: 00–063) and the Data Protection Authority. The researchers are indebted to the participants for their willingness to participate in the study.

Ethics permission for the LBC studies was obtained from the Multi-Centre Research Ethics Committee for Scotland (MREC/01/0/56) and from Lothian Research Ethics Committee (LBC1936: LREC/2003/2/29 and LBC1921: LREC/1998/4/183). The research was carried out in compliance with the Helsinki Declaration. All subjects gave written, informed consent.

## Additional files

Additional file 1: Table S1. Descriptive breakdown of each cohort and summary statistics. Additional file 2: Text S1. Additional Methods for each individual cohort. Additional file 3: Figure S1. Heterozygosity Metrics Determined Using Different SNP Lists. The dataset used was genome wide SNP data from sequencing of 503 individuals with European ancestry from 1000G phase 3 release. The SNP lists used were: 1) all SNPs 2) SNPs on the Illumina 1M 3) SNPs on the Illumina 610quad 4) SNPs on the Illumina Omni2.5 and 5) SNPs on the Affymetrix 6.0. This is to determine if SNP selection on the arrays biases the heterozygosity metric. We see high correlation and no systematic bias.
