# Supplementary material for: Genetic diversity is a predictor of mortality in humans
Source: BMC Genet. 2014 Dec 29;15:159. doi: 10.1186/s12863-014-0159-7 (PMC4301661; doi:10.1186/s12863-014-0159-7)
Supplement: Additional file 1: Table S1. — Descriptive breakdown of each cohort and summary statistics. [file 12863_2014_159_MOESM1_ESM.docx]

| **Study Name** | **AGES** | **ARIC** | **CHS** | **FHS** | **HealthABC** | **HRS** |
| --- | --- | --- | --- | --- | --- | --- |
| **Number Samples** | 3209 | 7825 | 3338 | 4526 | 1448 | 8617 |
| **Number Deaths** | 1021 | 1878 | 2220 | 1332 | 764 | 1089 |
| **Percent Female** | 58% | 53% | 60.3% | 54.90% | 45.86% | 58.22% |
| **Mean baseline Body Mass Index (BMI)** | 27.11 | 26.92 | N/A | 27.78 | 26.59 | 29.13 |
| **Mean followup time (Years)** | 7.66 | 19.39 | 13.4 | 9.14 | 10.93 | 4.64 |
| **Mean Age at Death (Years)** | 84.58 | 73.60 | 84.63 | 81.71 | 83.34 | 79.88 |
| **Years of baseline examinations** | 2002-2007 | 1987-1989 | 1989-1990 | 1974-2006 | 1997-1998 | 2006-2008 |
| **Years of DNA collection** | 2002-2007 | 1987-1998 | 1989-1990 | 1974-2006 | 1997-1998 | 2006-2008 |
| **Array type** | Illumina Hu370CNV | Affymetrix 6.0 | Illumina 370CNV | Affymetrix 500K mapping array, Affymetrix 50K supplemental gene-focused array | Illumina 1M | Illumina Human Omni2.5-4v1 |
| **Genotype calling algorithm** | BeadStudio | Birdseed | BeadStudio | BRLMM | GenomeStudio | GenomeStudio version 2011.2, Genotyping Module 1.9.4 and GenTrain version 1.0 |
| **Data handling and statistical tests** | PLINK and R | PLINK and R | PLINK and R | PLINK, R and SAS | PLINK and R | PLINK, R and SAS |
| **Education Level 1 (N)** | <11^th^ grade (769) | <11^th^ grade (1141) | <11^th^ grade (856) | <11^th^ grade (1315) | for those reporting grade 11 or less, also for those reporting vocation/trade school without GED (174) | <12th grade (1117) |
| **Education Level 2 (N)** | High school diploma, general equivalence diploma or some vocational school (1589) | High school diploma, general equivalence diploma or some vocational school (3686) | High school diploma, general equivalence diploma or some vocational school (1222) | High school diploma, general equivalence diploma or some vocational school (1788) | for high school graduates (497) | High school diploma, general equivalence diploma (4935) |
| **Education Level 3 (N)** | 1-4 years of college (487) | 1-4 years of college (2558) | 1-4 years of college (953) | 1-4 years of college (587) | some college (777) | 2yr college or 4yr college (1713) |
| **Education Level 4 (N)** | Some graduate/professional school (367) | Some graduate/professional school (790) | Some graduate/professional school (3381) | Some graduate/ professional school (303) | N/A | Master's degree or Ph.D. (852) |
| **Income Level 1 (N)** | N/A | Under $5000 (98) | Under $5000 (92) | N/A | for those reporting less than 10k (61) | Under $5000 (80) |
| **Income Level 2 (N)** | N/A | $5000-$7999 (118) | $5000-$7999 (216) | N/A | for 10k to 25k (463) | $5000-$7999 (106) |
| **Income Level 3 (N)** | N/A | $8000-$11999 (261) | $8000-$11999 (340) | N/A | for >25k up to <50k (581) | $8000-$11999 (378) |
| **Income Level 4 (N)** | N/A | $12000-$15999 (389) | $12000-$15999 (489) | N/A | for 50K+ (343) | $12000-$15999 (481) |
| **Income Level 5 (N)** | N/A | $16000-$24999 (1027) | $16000-$24999 (657) | N/A | N/A | $16000-$24999 (1017) |
| **Income Level 6 (N)** | N/A | $25000-$34999 (1541) | $25000-$34999 (539) | N/A | N/A | $25000-$34999 (1238) |
| **Income Level 7 (N)** | N/A | $35000-$49999 (1831) | $35000-$49999 (365) | N/A | N/A | $35000-$49999 (1373) |
| **Income Level 8 (N)** | N/A | Over $50000 (2578) | Over $50000 (492) | N/A | N/A | Over $50000 (3944) |
| **Linear Heterozygosity Whole Genome - Mean** | 0.9969 | 0.9989 | 0.9945 | 0.9973 | 0.9953 | 0.9987 |
| **Linear Heterozygosity Whole Genome - SD** | 0.0145 | 0.0096 | 0.0116 | 0.0096 | 0.0127 | 0.0086 |
| **Linear Heterozygosity Whole Genome - Max Number of SNPs Used** | 231171 | 371011 | 327843 | 211955 | 711732 | 840464 |

| **Study Name** | **INCHIANTI** | **LBC1921** | **LBC1936** | **MAP** | **ROS** | **Rotterdam** |
| --- | --- | --- | --- | --- | --- | --- |
| **Number Samples** | 1012 | 418 | 859 | 710 | 788 | 4903 |
| **Number Deaths** | 381 | 307 | 97 | 321 | 458 | 2751 |
| **Percent Female** | 55.14% | 56.22% | 47.85% | 72.41% | 65.68% | 59.36% |
| **Mean baseline Body Mass Index (BMI)** | 27.18 | 26.16 | 27.83 | 26.89 | 27.12 | 26.30 |
| **Mean followup time (Years)** | 11.36 | 5.12 | 2.98 | 6.09 | 9.69 | 13.22 |
| **Mean Age at Death (Years)** | 85.17 | 87.12 | 72.62 | 89.06 | 87.41 | 73.67 |
| **Years of baseline examinations** | 1998-2000 | 1999-2001 | 2004-2007 | 1997-2008 | 1994-2008 | 1990-1993 |
| **Years of DNA collection** | 1998-2000 | 1999-2001 | 2004-2007 | 1997-2008 | 1994-2008 | 1990-1993 |
| **Array type** | Illumina 550K | Illumina 610 quad v1 | Illumina 610 quad v1 | Affymetrix 6.0 | Affymetrix 6.0 | Illumina 550K |
| **Genotype calling algorithm** | Birdseed | Illumina GenomeStudio | Illumina GenomeStudio | Birdsuite, Broad Institute | Birdsuite, Broad Institute | The Beadstudio |
| **Data handling and statistical tests** | PLINK, R, SAS | PLINK and R | PLINK and R | PLINK and R | PLINK and R | PLINK and R |
| **Education Level 1 (N)** | <6th grade (692) | <11^th^ grade (285) | <11^th^ grade (627) | <11^th^ grade (50) | <11^th^ grade (18) | <11^th^ grade (1409) |
| **Education Level 2 (N)** | 6-12 years (179) | High school diploma, general equivalence diploma or some vocational school (50) | High school diploma, general equivalence diploma or some vocational school (133) | High school diploma, general equivalence diploma or some vocational school (227) | High school diploma, general equivalence diploma or some vocational school (38) | High school diploma, general equivalence diploma or some vocational school (2555) |
| **Education Level 3 (N)** | 12-16 years (98) | 1-4 years of college (50) | 1-4 years of college (48) | 1-4 years of college (213) | 1-4 years of college (42) | 1-4 years of college (1361) |
| **Education Level 4 (N)** | 16+ years (43) | Some graduate/professional school (33) | Some graduate/professional school (51) | Some graduate/professional school (397) | Some graduate/professional school (711) | N/A |
| **Income Level 1 (N)** | N/A | N/A | N/A | Under $5000 (15) | N/A | min-$23000 (760) |
| **Income Level 2 (N)** | N/A | N/A | N/A | $5000-$9999 (29) | N/A | >$23000-$32000 (840) |
| **Income Level 3 (N)** | N/A | N/A | N/A | $10000-$14999 (48) | N/A | >$32000-$40000 (869) |
| **Income Level 4 (N)** | N/A | N/A | N/A | $15000-$19999 (78) | N/A | >$40000-$52000 (912) |
| **Income Level 5 (N)** | N/A | N/A | N/A | $20000-$24999 (69) | N/A | >$52000-$70000 (1092) |
| **Income Level 6 (N)** | N/A | N/A | N/A | $25000-$34999 (135) | N/A | >$70000 (478) |
| **Income Level 7 (N)** | N/A | N/A | N/A | $35000-$49999 (145) | N/A | N/A |
| **Income Level 8 (N)** | N/A | N/A | N/A | Over $50000 (222) | N/A | N/A |
| **Linear Heterozygosity Whole Genome - Mean** | 0.9989 | 0.9991 | 1.0023 | 1.000 | 0.999 | 0.9980 |
| **Linear Heterozygosity Whole Genome - SD** | 0.0064 | 0.0087 | 0.0065 | 0.009 | 0.008 | 0.0070 |
| **Linear Heterozygosity Whole Genome - Max Number of SNPs Used** | 384883 | 410009 | 404427 | 297689 | 297689 | 433844 |

| **Study Name** | **SHIP** | **ARIC - African** | **HealthABC - African** | **HRS - African** | **CHS - African** |
| --- | --- | --- | --- | --- | --- |
| **Number Samples** | 3311 | 2473 | 995 | 1513 | 771 |
| **Number Deaths** | 424 | 825 | 584 | 220 | 562 |
| **Percent Female** | 44.85% | 62.38% | 55.58% | 63.85% | 62.13% |
| **Mean baseline Body Mass Index (BMI)** | 27.19 | 29.77 | 28.49 | 31.14 | N/A |
| **Mean followup time (Years)** | 11.23 | 18.58 | 10.10 | 4.52 | 11.95 |
| **Mean Age at Death (Years)** | 74.00 | 71.98 | 81.89 | 75.90 | 83.98 |
| **Years of baseline examinations** | 1997-2001 | 1987-1989 | 1997-1998 | 2006-2008 | 1989-1990 |
| **Years of DNA collection** | 1997-2001 | 1987-1998 | 1997-1998 | 2006-2008 | 1989-1990 |
| **Array type** | Affymetrix 6.0 | Affymetrix 6.0 | Illumina 1M | Illumina Human Omni2.5-4v1 | Illumina HumanOmni1-Quad_v1 |
| **Genotype calling algorithm** | Birdseed2 | Birdseed | GenomeStudio | GenomeStudio version 2011.2, Genotyping Module 1.9.4 and GenTrain version 1.0 | GenomeStudio |
| **Data handling and statistical tests** | PLINK and R | PLINK and R | PLINK and R | PLINK, R and SAS | PLINK and R |
| **Education Level 1 (N)** | <=8 years of school (1267) | <11^th^ grade (1028) | for those reporting grade 11 or less, also for those reporting vocation/trade school without GED (437) | <12th grade (534) | <11^th^ grade (336) |
| **Education Level 2 (N)** | 10 years of school (1475) | High school diploma, general equivalence diploma or some vocational school (736) | for high school graduates (301) | High school diploma, general equivalence diploma (730) | High school diploma, general equivalence diploma or some vocational school (210) |
| **Education Level 3 (N)** | >10 years of school (569) | 1-4 years of college (465) | some college (257) | 2yr college or 4yr college (174) | 1-4 years of college (142) |
| **Education Level 4 (N)** | N/A | Some graduate/professional school (370) | N/A | Master's degree or Ph.D. (75) | Some graduate/professional school (83) |
| **Income Level 1 (N)** | under 1375 €/month (582) | Under $5000 (340) | for those reporting less than 10k (264) | Under $5000 (61) | Under $5000 (112) |
| **Income Level 2 (N)** | - 1875 €/month (400) | $5000-$7999 (243) | for 10k to 25k (488) | $5000-$7999 (111) | $5000-$7999 (174) |
| **Income Level 3 (N)** | - 2375 €/month (452) | $8000-$11999 (302) | for >25k up to <50k (191) | $8000-$11999 (210) | $8000-$11999 (113) |
| **Income Level 4 (N)** | - 2875 €/month (491) | $12000-$15999 (268) | for 50K+ (52) | $12000-$15999 (169) | $12000-$15999 (111) |
| **Income Level 5 (N)** | - 3250 €/month (366) | $16000-$24999 (446) | N/A | $16000-$24999 (242) | $16000-$24999 (100) |
| **Income Level 6 (N)** | - 3750 €/month (290) | $25000-$34999 (305) | N/A | $25000-$34999 (198) | $25000-$34999 (84) |
| **Income Level 7 (N)** | - 4750 €/month (428) | $35000-$49999 (242) | N/A | $35000-$49999 (167) | $35000-$49999 (45) |
| **Income Level 8 (N)** | over 4750 €/month (302) | Over $50000 (176) | N/A | Over $50000 (355) | Over $50000 (32) |
| **Linear Heterozygosity Whole Genome - Mean** | 0.9991 | 0.9986 | 0.9968 | 0.9984 | 0.9958 |
| **Linear Heterozygosity Whole Genome - SD** | 0.0089 | 0.0156 | 0.0202 | 0.0120 | 0.0278 |
| **Linear Heterozygosity Whole Genome - Max Number of SNPs Used** | 389567 | 386600 | 757443 | 919711 | 654600 |
