# Supplementary material for: Genetic diversity is a predictor of mortality in humans
Source: BMC Genet. 2014 Dec 29;15:159. doi: 10.1186/s12863-014-0159-7 (PMC4301661; doi:10.1186/s12863-014-0159-7)
Supplement: Additional file 2: Text S1. — Additional Methods for each individual cohort. [file 12863_2014_159_MOESM2_ESM.docx]

**Supplemental**

**Figures**

Figure S1: Heterozygosity Metrics Determined Using Different SNP Lists

Also included as a supplement is a figure showing the relationship between heterozygosity metrics determined using different SNP lists [Additional File 3, Fig. S1]. The dataset used was genome wide SNP data from sequencing of 503 individuals with European ancestry from 1000G phase 3 release. The SNP lists used were: 1) all SNPs 2) SNPs on the Illumina 1M 3) SNPs on the Illumina 610quad 4) SNPs on the Illumina Omni2.5 and 5) SNPs on the Affymetrix 6.0. This is to determine if SNP selection on the arrays biases the heterozygosity metric. We see high correlation and no systematic bias.

**Tables**

Table S1: Cohort Demographics

**Methods - Cohorts**

**ARIC**

The Atherosclerosis Risk In Communities (ARIC) study[1] was established in 1986 as a prospective study of 15,792 individuals, 45-65 years of age, from 4 different US communities (Jackson, Mississippi; Forsyth County, North Carolina; Washington County, Maryland; suburbs of Minneapolis, Minnesota). The first visit was carried out in 1987-89, with four subsequent in-person visits and annual telephone interviews after initial visit. Mortality was tracked via telephone follow-ups, hospitalization records, state records, and the National Death Index. Each visit consisted of data collected about electrocardiographic measures and cardiovascular outcomes. Cause of death was determined using cause of death on the death certificate (ICD code). Only samples with a self-reported race of white or black were included in this analysis.

**AGES**

The Age, Gene/Environment Susceptibility (AGES Reykjavik) Study[2] was initiated to examine genetic susceptibility and gene/environment interaction as these contribute to phenotypes common in old age. The Reykjavik Study cohort originally comprised a random sample of 30,795 men and women born in 1907-1935 and living in Reykjavik in 1967. A total of 19,381 people attended, resulting in 71% recruitment rate. The study sample was divided into six groups by birth year and birth date within month. One group was designated for longitudinal follow up and was examined in all stages. One group was designated a control group and was not included in examinations until 1991. Other groups were invited to participate in specific stages of the study. Between 2002 and 2006, the AGES-Reykjavik study re-examined 5764 survivors of the original cohort who had participated before in the Reykjavik Study. Successful genotyping was available for 3219 AGES participants who were eligible for this study. The AGES Reykjavik Study GWAS was approved by the National Bioethics Committee and the Data Protection Authority.

**CHS**

The Cardiovascular Health Study (CHS)[3] is a population-based cohort study of risk factors for CHD and stroke in adults ≥65 years conducted across four field centers^1^. The original predominantly Caucasian cohort of 5,201 persons was recruited in 1989-1990 from random samples of the Medicare eligibility lists; subsequently, an additional predominantly African-American cohort of 687 persons were enrolled for a total sample of 5,888. DNA was extracted from blood samples drawn on all participants at their baseline examination in 1989-90. In 2007-2008, genotyping on the European ancestry individuals was performed at the General Clinical Research Center's Phenotyping/Genotyping Laboratory at Cedars-Sinai using the Illumina 370CNV BeadChip system on the CHS participants who were free of CVD at baseline, consented to genetic testing, and had DNA available for genotyping. In 2010, genotyping on the African-American CHS participants who consented to genetic testing, and had DNA available for genotyping was performed at the General Clinical Research Center's Phenotyping/Genotyping Laboratory at Cedars-Sinai using the Illumina HumanOmni1-Quad_v1 BeadChip system.

**FHS**

The Framingham Heart Study[4–7] is a community-based family study initiated to study determinants of cardiovascular and other chronic diseases. The study started in 1948 with the enrollment of 5,209 men and women who have been examined every two years since study inception. (1) In 1971, 5,124 children of the original cohort and spouses of the children were enrolled in the Offspring cohort and have been examined every 4 to 8 years.(2;3) Beginning in 2002, 4,095 adult grandchildren of the original cohort (children of the Offspring cohort) were enrolled into the Third Generation cohort (Gen 3) and have completed two examinations.(4) Blood samples for DNA collection were obtained on the surviving original cohort and Offspring members in the 1990s and on the Gen 3 participants on study entry. Participants from all three cohorts are under continuous surveillance for death. For the purposes of this genetic heterozygosity investigation, Original cohort and Offspring participants were followed from the time of DNA draw until death or end of follow –up in 2009. There were 4525 participants (2485 women) included in the study sample and 1332 deaths (664 deaths in women, mean age at death 81.7 years) occurred during follow-up (mean follow –up time 9.1 years). Cox proportional hazards models were used to determine the linear effect of heterozygosity on survival. A robust variance estimate clustering on family was used to account for family correlation. Additional models examined the association of quintiles of heterozygosity with survival. As described in the analysis section, the models were adjusted for body mass index at baseline and principal components 1-10, stratified by sex and education category. In secondary analyses, sex specific models and by-chromosome heterozygosity were examined. Income at baseline was not available and therefore not included in the model.

**HealthABC**

HealthABC[8]. Genomic DNA was extracted from buffy coat collected using PUREGENE® DNA Purification Kit during the baseline exam. In 2009, genotyping was performed by the Center for Inherited Disease Research (CIDR) using the Illumina Human1M-Duo BeadChip system. Samples were excluded from the dataset for the reasons of sample failure (call rate < 95%), genotypic sex mismatch, and first-degree relative of an included individual based on genotype data. African American and European ancestry were confirmed using principal components analyses with HapMap 3 populations as references. Genotyping was successful in 2,802 individuals (1663 European ancestry and 1139 African Americans). Genotypes were available on 914263 high quality SNPs.

**HRS**

The Health and Retirement Study (HRS)[9] is a longitudinal survey of a representative sample of Americans over the age of 50. The current sample is over 26,000 persons in 17,000 households. The study interviews respondents every two years about income and wealth, health and use of health services, work and retirement, and family connections. DNA was extracted from saliva collected during a face-to-face interview in the respondents' homes. These data represent respondents who provided DNA samples and signed consent forms in 2006 and 2008.

**InCHIANTI**

The InCHIANTI study[10] is a population-based epidemiological study aimed at evaluating the factors that influence mobility in the older population living in the Chianti region in Tuscany, Italy. The details of the study have been previously reported. Briefly, 1616 residents were selected from the population registry of Greve in Chianti (a rural area: 11,709 residents with 19.3% of the population greater than 65 years of age), and Bagno a Ripoli (Antella village near Florence; 4,704 inhabitants, with 20.3% greater than 65 years of age). The participation rate was 90% (n=1453), and the subjects ranged between 21-102 years of age. Illumina Infinium HumanHap 550K SNP arrays were used for genotyping. The study protocol was approved by the Italian National Institute of Research and Care of Aging Institutional Review and Medstar Research Institute (Baltimore, MD).

**LBC**

Lothian Birth Cohorts 1921 and 1936 (LBC1921, LBC1936)[11–13]

The Lothian Birth Cohort 1936 (LBC1936) consists of 1,091 relatively healthy individuals assessed on cognitive and medical traits at about 70 years of age. They were born in 1936, most took part in the Scottish Mental Survey of 1947, and almost all lived independently in the Lothian region of Scotland in older age. At recruitment the sample of 548 men and 543 women had a mean age 69.6 years (SD = 0.8). Two further waves were carried out at 73 (n = 866 (448 males, 418 females) and 76 years of age (in progress). A full description of participant recruitment and testing can be found elsewhere (Deary et al 2011, 2007). At recruitment the LBC1921 cohort consisted of 550 relatively healthy individuals, 316 females and 234 males, assessed on cognitive and medical traits at about 79 years of age. Three further testing waves were completed at 83 (n = 321 (145 males, 176 females), 87 (n = 235 (109 males, 126 females) and 90 (n = 129 (59 males, 70 females) years of age. They were all born in 1921, most took part in the Scottish Mental Survey of 1932, and almost all lived independently in the Lothian region (Edinburgh City and surrounding area) in Scotland in older age. When tested, the sample had a mean age of 79.1 years (SD = 0.6). A full description of participant recruitment and testing can be found elsewhere (Deary et al 2011, 2004). Ethics permission for the study was obtained from the Multi-Centre Research Ethics Committee for Scotland (MREC/01/0/56) and from Lothian Research Ethics Committee (LBC1936: LREC/2003/2/29 and LBC1921: LREC/1998/4/183). The research was carried out in compliance with the Helsinki Declaration. All subjects gave written, informed consent.

**MAP/ROS**

Religious Orders Study (ROS)[14]

The ROS, started in 1994, is a longitudinal, clinical-pathologic cohort study of common chronic conditions of aging. The study enrolls Catholic priests, nuns, and brothers from about 40 groups in 12 states of the United States. Since January 1994, over 1,100 participants completed their baseline evaluation. The study was approved by the institutional review board of Rush University Medical Center. The follow-up rate of survivors exceeds 90%. Participants were free of known dementia at enrollment, agreed to annual clinical evaluations, and signed both an informed consent and an Anatomic Gift Act form donating their brains at time of death. DNA was extracted from whole blood, lymphocytes, or frozen postmortem brain tissue. Genotyping was performed at the Broad Institute’s Center for Genotyping and the Translational Genomics Research Institute.

Rush Memory and Aging Project (MAP)[15]

The MAP, started in 1997, is a longitudinal, clinical-pathologic cohort study of common chronic conditions of aging. The study enrolled older men and women from assisted living facilities in the Chicago area with no evidence on dementia at baseline. Since October 1997, over 1,500 participants completed their baseline evaluation. The study was approved by the institutional review board of Rush University Medical Center. The follow-up rate of survivors exceeds 90%. Similar to the ROS, participants agreed to annual clinical evaluations and signed both an informed consent and an Anatomic Gift Act form donating their brains, spinal cords, and selected nerves and muscles to Rush investigators at the time of death. DNA was extracted from whole blood, lymphocytes, or frozen postmortem brain tissue. Genotyping was performed at the Broad Institute’s Center for Genotyping and the Translational Genomics Research Institute.

**Rotterdam**

The Rotterdam Study[16] is a prospective cohort study ongoing since 1990 in the city of Rotterdam in The Netherlands. The study targets cardiovascular, endocrine, hepatic, neurological, ophthalmic, psychiatric, dermatological, oncological, and respiratory diseases. As of 2008, 14,926 subjects aged 45 years or over comprise the Rotterdam Study cohort. The findings of the Rotterdam Study have been presented in over a 1,000 research articles and reports ([www.erasmus-epidemiology.nl/rotterdamstudy](http://www.erasmus-epidemiology.nl/rotterdamstudy)).

**SHIP**

The Study of Health in Pomerania (SHIP)[17, 18] is a cross-sectional survey in West Pomerania, the north-east area of Germany. A sample from the population aged 20 to 79 years was drawn from population registries. First, the three cities of the region (with 17,076 to 65,977 inhabitants) and the 12 towns (with 1,516 to 3,044 inhabitants) were selected, and then 17 out of 97 smaller towns (with less than 1,500 inhabitants), were drawn at random. Second, from each of the selected communities, subjects were drawn at random, proportional to the population size of each community and stratified by age and gender. Only individuals with German citizenship and main residency in the study area were included. Finally, 7,008 subjects were sampled, with 292 persons of each gender in each of the twelve five-year age strata. In order to minimize drop-outs by migration or death, subjects were selected in two waves. The net sample (without migrated or deceased persons) comprised 6,267 eligible subjects. Selected persons received a maximum of three written invitations. In case of non-response, letters were followed by a phone call or by home visits if contact by phone was not possible. The SHIP population finally comprised 4,308 participants (corresponding to a final response of 68.8%).

Genotyping

The SHIP samples were genotyped using the Affymetrix Genome-Wide Human SNP Array 6.0. Hybridisation of genomic DNA was done in accordance with the manufacturer’s standard recommendations. The genetic data analysis workflow was created using the Software InforSense. Genetic data were stored using the database Caché (InterSystems). Genotypes were determined using the Birdseed2 clustering algorithm. For quality control purposes, several control samples where added. On the chip level, only subjects with a genotyping rate on QC probesets (QC callrate) of at least 86% were included. Finally, all arrays had a sample callrate > 92%. The overall genotyping efficiency of the GWA was 98.55%. Duplicate samples as estimated by IBD and individuals with mismatch between reported and genotyped gender were excluded from analysis.

1. The Atherosclerosis Risk in Communities (ARIC) Study: design and objectives. The ARIC investigators. *Am J Epidemiol* 1989, 129:687–702.

2. Harris TB, Launer LJ, Eiriksdottir G, Kjartansson O, Jonsson PV, Sigurdsson G, Thorgeirsson G, Aspelund T, Garcia ME, Cotch MF, Hoffman HJ, Gudnason V: Age, Gene/Environment Susceptibility-Reykjavik Study: multidisciplinary applied phenomics. *Am J Epidemiol* 2007, 165:1076–1087.

3. Fried LP, Borhani NO, Enright P, Furberg CD, Gardin JM, Kronmal RA, Kuller LH, Manolio TA, Mittelmark MB, Newman A: The Cardiovascular Health Study: design and rationale. *Ann Epidemiol* 1991, 1:263–276.

4. DAWBER TR, MEADORS GF, MOORE FE Jr: Epidemiological approaches to heart disease: the Framingham Study. *Am J Public Health Nations Health* 1951, 41:279–281.

5. Feinleib M, Kannel WB, Garrison RJ, McNamara PM, Castelli WP: The Framingham Offspring Study. Design and preliminary data. *Prev Med* 1975, 4:518–525.

6. Kannel WB, Feinleib M, McNamara PM, Garrison RJ, Castelli WP: An investigation of coronary heart disease in families. The Framingham offspring study. *Am J Epidemiol* 1979, 110:281–290.

7. Splansky GL, Corey D, Yang Q, Atwood LD, Cupples LA, Benjamin EJ, D’Agostino RB Sr, Fox CS, Larson MG, Murabito JM, O’Donnell CJ, Vasan RS, Wolf PA, Levy D: The Third Generation Cohort of the National Heart, Lung, and Blood Institute’s Framingham Heart Study: design, recruitment, and initial examination. *Am J Epidemiol* 2007, 165:1328–1335.

8. Harris TB, Visser M, Everhart J, Cauley J, Tylavsky F, Fuerst T, Zamboni M, Taaffe D, Resnick HE, Scherzinger A, Nevitt M, For the Health A, Study BC: Waist Circumference and Sagittal Diameter Reflect Total Body Fat Better Than Visceral Fat in Older Men and Women: The Health, Aging and Body Composition Study. *Ann N Y Acad Sci* 2000, 904:462–473.

9. Juster FT, Suzman R: An Overview of the Health and Retirement Study. *J Hum Resour* 1995, 30:S7–S56.

10. Ferrucci L, Bandinelli S, Benvenuti E, Di Iorio A, Macchi C, Harris TB, Guralnik JM: Subsystems contributing to the decline in ability to walk: bridging the gap between epidemiology and geriatric practice in the InCHIANTI study. *J Am Geriatr Soc* 2000, 48:1618–1625.

11. Deary IJ, Whiteman MC, Starr JM, Whalley LJ, Fox HC: The impact of childhood intelligence on later life: following up the Scottish mental surveys of 1932 and 1947. *J Pers Soc Psychol* 2004, 86:130–147.

12. Deary IJ, Gow AJ, Taylor MD, Corley J, Brett C, Wilson V, Campbell H, Whalley LJ, Visscher PM, Porteous DJ, Starr JM: The Lothian Birth Cohort 1936: a study to examine influences on cognitive ageing from age 11 to age 70 and beyond. *BMC Geriatr* 2007, 7:28.

13. Deary IJ, Gow AJ, Pattie A, Starr JM: Cohort profile: the Lothian Birth Cohorts of 1921 and 1936. *Int J Epidemiol* 2012, 41:1576–1584.

14. Bennett DA, Schneider JA, Arvanitakis Z, Wilson RS: Overview and findings from the religious orders study. *Curr Alzheimer Res* 2012, 9:628–645.

15. Bennett DA, Schneider JA, Buchman AS, Barnes LL, Boyle PA, Wilson RS: Overview and findings from the rush Memory and Aging Project. *Curr Alzheimer Res* 2012, 9:646–663.

16. Hofman A, Breteler MMB, van Duijn CM, Krestin GP, Pols HA, Stricker BHC, Tiemeier H, Uitterlinden AG, Vingerling JR, Witteman JCM: The Rotterdam Study: objectives and design update. *Eur J Epidemiol* 2007, 22:819–829.

17. John U, Greiner B, Hensel E, Lüdemann J, Piek M, Sauer S, Adam C, Born G, Alte D, Greiser E, Haertel U, Hense HW, Haerting J, Willich S, Kessler C: Study of Health In Pomerania (SHIP): a health examination survey in an east German region: objectives and design. *Soz- Präventivmedizin* 2001, 46:186–194.

18. Völzke H, Alte D, Schmidt CO, Radke D, Lorbeer R, Friedrich N, Aumann N, Lau K, Piontek M, Born G, Havemann C, Ittermann T, Schipf S, Haring R, Baumeister SE, Wallaschofski H, Nauck M, Frick S, Arnold A, Jünger M, Mayerle J, Kraft M, Lerch MM, Dörr M, Reffelmann T, Empen K, Felix SB, Obst A, Koch B, Gläser S, et al.: Cohort profile: the study of health in Pomerania. *Int J Epidemiol* 2011, 40:294–307.
