# Supplementary figures and images for: Genetic diversity is a predictor of mortality in humans
Source: BMC Genet. 2014 Dec 29;15:159. doi: 10.1186/s12863-014-0159-7 (PMC4301661; doi:10.1186/s12863-014-0159-7)

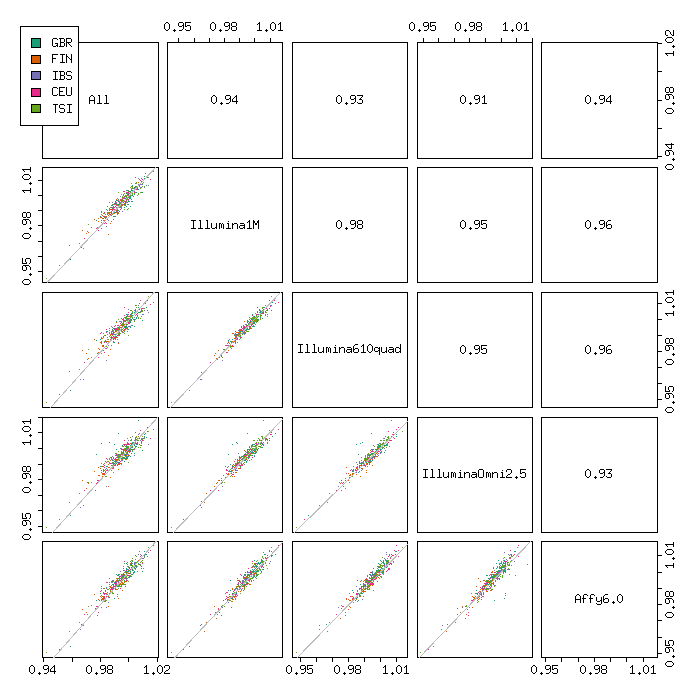

Supplement: Additional file 3: Figure S1. — Heterozygosity Metrics Determined Using Different SNP Lists. The dataset used was genome wide SNP data from sequencing of 503 individuals with European ancestry from 1000G phase 3 release. The SNP lists used were: 1) all SNPs 2) SNPs on the Illumina 1M 3) SNPs on the Illumina 610quad 4) SNPs on the Illumina Omni2.5 and 5) SNPs on the Affymetrix 6.0. This is to determine if SNP selection on the arrays biases the heterozygosity metric. We see high correlation and no systematic bias. [file 12863_2014_159_MOESM3_ESM.png]
